# Reviews

**Published:** 1896-08

**Authors:** 


### REVIEWS.

The well-known house of W. R. Jenkins, 853 Sixth Avenue, New York City, which for so many years has taken the lead in the publication of veterinary works, and which in so doing has contributed so much to the true growth of the veterinary profession, has again favored us by presenting the recent translation by Prof. A. Liautard of C. Pellerin's contribution relative to the value of *Median Neurotomy in the Treatment of Chronic Tendonitis and Periostitis of the Fetlock*. The translation bears with it the experience and ripe judgment of Prof. Liautard, whose rank as a surgeon is not surpassed in our country. The work will be gratefully received by those veterinarians who for many years have relied upon counter-irritation and the actual cautery for dealing with these lesions, and who have had so many grievous disappointments in this line of treatment. The operation has already found favor in this country, the July number of the Journal recording a series of favorable results by one operator. Dr. Pellerin was formerly connected with the Alfort Veterinary School.

Another valuable work to the busy practitioner and student has been issued by the same house. It is entitled *Outlines of Veterinary Anatomy*. Part I. covers the anterior and posterior limbs, by Prof. O. Charnock Bradley, of the New Veterinary College, Edinburgh, Scotland. Illustrated by many diagrams, the text is thus made clearer to the student and the subject more readily and quickly grasped. To the practitioner about to enter the operating arena it will promptly afford information upon which he may be a little uncertain, and will quickly mirror the field of his operation before him. Typographically it is a very attractive product of the printer's art.

Mr. Jenkins has also issued, for the use of lecturers, demonstrators, and others specially engaged in teaching, a series of diagrams of the horse, the work of Prof. Sussdorf, M.D., and translated into English by Prof. W. Owen Williams, of the New Veterinary College, Edinburgh, Scotland. These diagrams are shortly to be followed by similar ones of the cow.

Surely with these many valuable aids the future student, and later the veterinarian, must become in every way better equipped for his work and more thoroughly fitted to pursue his vocation. With increased facilities and increased time to take advantage of the same, the future graduate must be of much more worth to his community, and be better prepared in every way than was his predecessor to promptly deal with the perplexing questions and demands of his profession.

*Gould's Students' Medical Dictionary*. Tenth edition. Messrs. P. Blakiston, Son & Co., 1012 Walnut Street, Philadelphia. This book, typical of the printer's highest art and up to date in its contents, will be of special interest at this time to prospective veterinary and medical students. Intending matriculants who purpose doing some preparatory reading will find it of the greatest value to them, and the busy student at college should have it continually at his right hand. Replete with information, valuable tables and lists, it will continue to find in the future, as other editions have in the past, a wide sale and very appreciative readers to daily consult its pages.

Veterinarian A. C. Haasloch, Lecturer on Materia Medica and Therapeutics at the New York College of Veterinary Surgeons, has brought forth, through the well-known publishing house of W. R. Jenkins, a *Veterinary Compend of Materia Medica and Therapeutics*. The little volume is neat and convenient in size and form, and printed in clear, bold type. It briefly covers the whole subject of materia medica, going into no detail of the matter presented. It will prove a valuable reference-book to the student, and aid him in his studies and preparation for examination; alike it will find a place on the office table or pharmacy shelf of the busy veterinarian, where its prompt aid will suffice for a time the busy worker when one is in doubt. The arrangement of the various drugs under the chief action they possess makes it more attractive and presentable. The veterinarian has lacked in his field many of these assistants, and so has the student at the end of his college term when reviewing for examinations, and they will find in this work a help in time of need.

On to Buffalo!

